# Combined Coal and Gas Kitchener

**Published:** 1915-08-14

**Authors:** 


*To the Editor of* The Hospital.

### Combined Coal and Gas Kitchener.

*To the Editor of* The Hospital :

*Bureau of Information.*

Sir,—I note among the answers in the *Institutional Worker* Supplement of last week a reference to a combined coal and gas cooking range. As I use one of these kitcheners I at once examined it for the name of the makers, which is the same as that given in your paragraph. A reference to my experience might be of interest, as I believe these ranges are not at all common.

The oven is, of course, the only part which might cause trouble to a cook; the rings and griller on the top differ in no way from those on any ordinary gas cooker. One trouble, though not a constant one, is that the burners around the base of the oven are apt to go out. If they all go out, the flue is probably to blame, but sometimes a few go out for no apparent reason, and then the escaping gas is liable to become a source of danger or to taint the meat unless the oven is constantly watched.

These burners, in the case of my particular range, differ from those usually found in gas ovens. There are several nipples with three holes inserted at intervals on the top of the gas-pipe, instead of the usual lateral holes; and the flames are yellow instead of the blue aërated flames of the ordinary gas ring. What the advantage of this is I do not see, and it would be interesting to know if this is correct or if there is some defect. As for its cooking abilities, there is no cause to grumble when the oven is working well.—Yours faithfully, M. A. M.

August 7, 1915.

[Apparently the air-inlet hole which ordinarily produces the Bunsen flame has become choked with soot or in some manner which produces the defect referred to. Has our correspondent had the gas section of his combined kitchener lately cleaned?—Ed. The Hospital.]

